# Effect of “motivational interviewing” and “information, motivation, and behavioral skills” counseling interventions on choosing the mode of delivery in pregnant women: a study protocol for a randomized controlled trial

**DOI:** 10.1186/s13063-020-04865-3

**Published:** 2020-11-25

**Authors:** Mahboubeh Shirzad, Elham Shakibazadeh, Abbas Rahimi Foroushani, Mehrandokht Abedini, Hamid Poursharifi, Sohrab Babaei

**Affiliations:** 1 Department of Health Education and Promotion, School of Public Health, Tehran University of Medical Sciences, Tehran, Iran; 2 Department of Epidemiology and Biostatistics, School of Public Health, Tehran University of Medical Sciences, Tehran, Iran; 3 Maternal Health Department, Ministry of Health, Tehran, Iran; 4 Department of Psychology, University of Social Welfare and Rehabilitation Sciences, Tehran, Iran

**Keywords:** Cesarean section, Normal vaginal delivery, Motivational interviewing, Information, motivation, and behavioral skills, Iran

## Abstract

**Background:**

Cesarean section is an important surgical procedure, when normal vaginal delivery imposes a risk to mother and/or baby. The World Health Organization states the ideal rate for Cesarean section to be between 10 and 15% of all births. In recent decades, the rate has been increased dramatically worldwide. This paper explains the protocol of a randomized controlled trial that aims to compare the effect of “motivational interviewing” and “information, motivation, and behavioral skills” counseling interventions on choosing mode of delivery in pregnant women.

**Methods:**

A four-armed, parallel-design randomized controlled trial will be conducted on pregnant women. One hundred and twenty women will be randomly assigned to four groups including three intervention groups and one control group. The intervention groups included the following: (1) motivational interviewing; (2) face-to-face information, motivation, and behavioral skills model; and (3) information, motivation, and behavioral skills model provided using a mobile application. The inclusion criteria include being literate, being in gestational age from 24 to 32 weeks, being able to speak Persian, having no complications in the current pregnancy, having no indications for Cesarean section, and having enough time to participate in the intervention. The primary outcome of the study is the mode of delivery. The secondary outcomes are women’s intention to undergo Cesarean section and women’s self-efficacy.

**Discussion:**

The interventions of this protocol have been programmed to reduce unnecessary Cesarean sections. Findings may contribute to a rise in normal vaginal delivery, and the effective intervention may be extended for use in national Cesarean section plans.

**Trial registration:**

Iran Randomized Clinical Trial Center IRCT20151208025431N7. Registered on December 07, 2018.

## Administrative information

The order of the items has been modified to group similar items (see http://www.equator-network.org/reporting-guidelines/spirit-2013-statement-defining-standard-protocol-items-for-clinical-trials/).

**Table:** 

Title {1}	Effect of “motivational interviewing” and “information, motivation, and behavioral skills” counseling interventions on choosing the mode of delivery in pregnant women: a study protocol for a randomized controlled trial
Trial registration {2a and 2b}.	This study has been registered in Iran Randomized Clinical Trial Center (IRCT20151208025431N7). Registered December 07, 2018.
Protocol version {3}	V2.0, 1st December 2019.
Funding {4}	This study is being financially supported by Tehran University of Medical Sciences (TUMS) and Iran National Science Foundation (INSF).
Author details {5a}	Mahboubeh Shirzad^1^, Elham Shakibazadeh^1^, Abbas Rahimi Foroushani^2^, Mehrandokht Abedini^3^, Hamid Poursharifi^4^, Sohrab Babaei^1^ 1. Department of Health Education and Promotion, School of Public Health, Tehran University of Medical Sciences, Tehran, Iran. 2. Department of Epidemiology and Biostatistics, School of Public Health, Tehran University of Medical Sciences, Tehran, Iran. ORCID number: 0000-0002-3052-6420 3. Maternal Health Department, Ministry of Health, Tehran, Iran 4. Department of Psychology, University of Social Welfare and Rehabilitation Sciences, Tehran, Iran. ORCID ID: 0000-0003-3864-9924 Correspondence: Elham Shakibazadeh, ORCID ID: 0000-0002-1320-2133
Name and contact information for the trial sponsor {5b}	Tehran University of Medical Sciences (TUMS); email: vcr@tums.ac.ir Iran National Science Foundation (INSF); e-mail: info@insf.org
Role of sponsor {5c}	The role of the funders is to monitor the corresponding research planning and progression.

## Introduction

### Background and rationale {6a}

Cesarean section (CS) is an important surgical procedure, when normal vaginal delivery (NVD) imposes a risk to mother and/or baby [1]. The World Health Organization (WHO) states that a rate of CS between 10 and 15% of all births is ideal; however, the rate is steadily growing in recent years [2]. The average worldwide rate of CS is 18.6%, ranging from 6.0 to 27.2% in the least and more developed countries, respectively. Countries with the highest CS rates are Brazil (55.6%) and Dominican Republic (56.4%) in Latin America and the Caribbean. Iran and Turkey (47.9% and 47.5%, respectively) have the highest rates in Asia [3]. In Iran, the rate is even higher in private hospitals (72–89%) [4–7].

CS can save the lives of mothers and infants in emergency situations. However, current rates suggest that the CS is now used for women with normal and non-complicated pregnancies and births, when it is not medically necessary [8]. Unnecessary CSs could create complications with no benefits to both mother and baby [3, 9–12]. According to an observational study conducted by the WHO in nine Asian countries, women who undergo an unplanned CS before or during labor or who have an assisted (operative) vaginal delivery are more likely to experience morbidity than those who have spontaneous vaginal delivery [13]. As with any surgery, CS is associated with short- and long-term risks that may be minor or severe [14]. CS can be associated with significant short-term risks such as asphyxia, if the uterus is hypoperfused due to anesthesia, scalpel lacerations, and neonatal respiratory morbidities [9]. Other short-term risks of CS include increased risk of infection and lower likelihood of breastfeeding [10, 11, 15]. Moreover, urinary catheterization is associated with post-CS bacteriuria and has been reported to be as high as 11% [16]. Increasing rates of CS are associated with increased maternal and perinatal morbidities [17].

In 2014, Iran’s “health sector evolution policy” was launched to improve public health. One important objective of this policy was to decrease the rate of unnecessary CSs [18]. Several strategies have been conducted such as freeing NVDs in all public hospitals, developing mother-friendly hospitals, developing standard protocols of birth and preparation classes for women, improving privacy and infrastructure of labor, promoting standards in birth facilities, promoting water birth, and determining financial incentives to doctors to encourage them to do NVDs in public hospitals [19]. There was a reduction in CS rate after implementing the policy; however, the rate is still significantly higher than the rate recommended by the WHO [19].

Several studies have been conducted on reasons behind the high rate of CS in Iran [20–23]. Studies have shown that the main reason is the willingness of women to undergo CS due to fear of pain during labor and childbirth [21, 24, 25], concerns about genital modifications after vaginal delivery [26–28], belief that CS is safer for the baby [29–31], and the convenience for women and their families [18]. Studies show that women can play a major role in the decision-making process about their birth [32–36].

In recent years, different interventions intended to reduce the CS rate in Iran [37–40]. Although these interventions have been effective in the short term, they have not been effective in the long term. To further reduce the rate of CS, it is necessary not only to address the health system, health facility, and health professional factors, but also to change women’s choice behaviors [19]. The WHO has provided recommendations on non-clinical interventions to reduce unnecessary CSs. The recommendations are grouped according to the target of intervention: (a) interventions targeted at women, (b) interventions targeted at healthcare professionals, and (c) interventions targeted at health organizations, facilities, or systems [41].

Regarding non-clinical interventions on reducing unnecessary CS targeted at women, the WHO has recommended implementing psycho-education interventions for women [8, 42]. A Cochrane review conducted by Chen et al. on non-clinical interventions for reducing unnecessary CS reported that psycho-education interventions were effective in reducing unnecessary CSs [43]. The educational interventions included psycho-education on fear of childbirth [44], intensive group therapy (cognitive-behavioral therapy and childbirth psycho-therapy) [45], psycho-education by telephone [46], role-play education versus standard education using lectures [47], and nurse-led applied relaxation training program [44].

Several psycho-educational models and methods/strategies have been introduced to change behaviors effectively. Motivational interviewing (MI) is a patient-centered counseling approach to motivate individuals to change their behaviors [48], and it is specifically designed to enhance motivation to change among patients not ready to change [49]. It highlights the importance of motivation in personal behavior change. Research on MI has demonstrated positive effects of helping patients clarify goals, explore obstacles to treatment, and make commitments to change [49]. MI is a relatively new cognitive-behavioral technique that aims to help patients identify and change behaviors that may be placing them at risk of developing health problems or may be preventing optimal management of a chronic condition.

In the Information-Motivation-Behavioral skills (IMB) model, preventive behavioral skills represent a final common pathway for predicting complex preventive behaviors [50]. The IMB model is a generalizable and simple model to guide thinking about complex health behaviors. The IMB constructs, and how they pertain to patient adherence, are outlined below: (1) *Information* is the basic knowledge about a medical condition that might include how the disease develops, its expected course, and effective strategies for its management; (2) *Motivation* encompasses personal attitudes towards the adherence behavior, the perceived social support for such behavior, and the patients’ subjective norms or perceptions of how others with this medical condition might behave; and (3) *Behavioral skills* include ensuring that the patient has specific behavioral tools or strategies necessary to perform the adherence behavior such as enlisting social support and other self-regulation strategies.

In recent years, mobile applications play an important role in delivering educational content. People carry their mobile phones with themselves wherever they go, so educational interventions can be delivered at any time to anyone with extra support upon request wherever and whenever it is needed. This opportunity provides simple and non-expensive interventions to various ranges of individuals. Motivational messages, monitoring, and behavior change tools can be modified for delivery via mobile phones [51]. The effectiveness of this type of intervention is affirmed in several studies such as smoking cessation [52], adherence to prescribed medication [53], and blood pressure management [54]. This paper explains our study protocol aiming at comparing the effect of MI and IMB, and IMB based on mobile application (IMB-App) on choosing the mode of delivery in pregnant women.

### Objectives {7}

This is the protocol of our study that aims at comparing the effect of IM, IMB, and IMB-App counseling interventions on choosing the mode of delivery in pregnant women.

### Trial design {8}

This study is a randomized controlled, parallel-design trial.

## Methods: participants, interventions, and outcomes

### Study setting {9}

Pregnant women will be chosen from private hospitals located in Tehran. We have chosen private hospitals, because they support elective CS by providing high-quality facilities for women. Only in private hospitals, women are able to choose CS as a mode of delivery (maternal request) [18].

### Eligibility criteria {10}

#### Inclusion criteria

The criteria for entering women are being literate, being in gestational age 24 to 32 weeks, being able to speak Persian, having no complications in the current pregnancy, having no indications for CS, and having enough time to participate in the interventions.

#### Exclusion criteria

We exclude women who show complications during the study, have preterm labor, and are reluctant to continue to participate in this study.

### Who will take informed consent? {26a}

Written informed consent will be obtained from all the participants by the principal investigator or sub-investigators prior to enrollment.

### Additional consent provisions for collection and use of participant data and biological specimens {26b}

This is not applicable. No biological specimens will be collected for research purposes; hence, no additional consent will be sought from the participants.

## Interventions

### Explanation for the choice of comparators {6b}

This is not applicable.

### Intervention description {11a}

1. The content of interventions will be designed based on a qualitative evidence synthesis and a quantitative systematic review and meta-analysis in Iran [55]. Also, we will design the interventions tailored to the participants’ needs. The interventions are expected to take around 2 months to be completed, and women will be followed up until their delivery time.
2. Motivational interviewing (MI)
  In this intervention group, pregnant women will be trained face-to-face based on the MI. During three 45–60-min sessions, MI techniques will be provided to the participants. MI is a directive, client-centered counseling style for eliciting behavior change by helping clients to explore and resolve ambivalence. It is most centrally defined not by technique but by its spirit as a facilitative style for interpersonal relationships. MI is a relatively simple, transparent, and supportive talk therapy based on the principles of cognitive-behavior therapy. In this group, we will help women to explore and resolve ambivalence about the mode of delivery and build their intrinsic motivation.
  We will not force women to choose a specific mode of delivery. We will ask open-ended questions (for example, “Tell me what you think about CS?” or “What do encourage you to choose this type of delivery (CS or NVD)?”). Open-ended questions can help us to understand how they are thinking about the mode of delivery. Affirming is one of the fundamental MI skills. We will use it to support engagement, encourage the women to further explore change processes, and build confidence. We will find an opinion the client is making or a strength she will notice and reflect it to her (for example, “So how did you manage to control your fear after attending our training sessions?”). We will use reflective listening as a simple method to reduce resistance in MI. The last step of this technique is to summarize what the pregnant women have said.
3. Information, motivation, and behavioral skills model through a face-to-face approach (IMB)
  During three 45–60-min sessions, the model’s strategies will be provided to the participants. Participants will receive information and behavioral skills related to the choice of mode of delivery as well as internal and external motivational factors related to the choice of delivery. The strategy includes the following: (1) *Information*—the intervention will begin with information on the prevalence of CS and CS-related complications in women, and outcome of unnecessary CS; (2) *Motivation*—the interventionist will perform this technique to motivate pregnant women, providing personal feedback, asking open-ended questions, affirming desirable behavior, reflective listening, working at the women’s pace, and negotiating goals that will be realistic and attainable; and (3) *Behavioral skills*—women will be given behavioral skills training on how to control the obstacle of NVD. To build skills for choosing the mode of delivery, training will be given on how to reduce these barriers.
4. Information, motivation, and behavioral skills model through mobile app (IMB-App)
  The mobile application (M-health) has been designed based on the IMB model. The software will be installed on mobile phones of participants in the group, and its operation will be taught individually. Women will work with the application in the presence of the research team, and any existing problems will be resolved. The strategies foreseen for adherence improvement include reminders set at defined intervals in the form of pop-up messages. To monitor adherence, the data collected on the server will be used. In addition to the application, a server will be designed in which the users’ activities will be collected. Items such as the duration of application usage and the sections used by the user (in addition to registering their time and duration) will be registered. Every time the user’s mobile is connected to the Internet, the data will be uploaded and saved on to the server. These data can be used as a proxy of adherence to the intervention.

*Control group*: without any intervention

### Criteria for discontinuing or modifying allocated interventions {11b}

The intervention will be discontinued based on the participant’s request, or if a woman undergoes birth.

### Strategies to improve adherence to interventions {11c}

This is not applicable.

### Relevant concomitant care permitted or prohibited during the trial {11d}

Usual prenatal care will be provided in all study groups.

### Provisions for post-trial care {30}

This is not applicable.

### Outcomes {12}

- Determining and comparing the effects of MI, IMB, and IMB-App on mode of delivery in the target and control groups before and after the interventions.
- Determining and comparing the effects of MI, IMB, and IMB-App on the intention of performing CS in women in the target and control groups before and after the interventions.
- Determining and comparing the effect of MI, IMB, and IMB-App on women’s self-efficacy in target and control groups before and after the interventions.

### Participant timeline {13}

The recruitment of participants for this study started in December 2019 and is still ongoing. The recruitment is expected to be completed in April 2020; the data analysis, writing of scientific manuscripts, and submissions to peer-reviewed scientific journals will occur from 2020 to 2021.

### Sample size {14}

The sample size is 120 pregnant women (30 in each group) with a power of 80% to detect a minimum difference.

### Recruitment {15}

We will be present in the research setting during the intervention period to recruit all eligible women. The study will be available to all pregnant women interested in participating and speaking Persian. Individuals who fulfill the inclusion criteria will receive a description of the study, indicating the follow-up schedule and assurance of confidentiality, and they will be directed to complete a consent form. Included participants will be randomly allocated in three intervention groups and one control group.

## Assignment of interventions: allocation

### Sequence generation {16a}

The participants will be randomly assigned to four groups after the initial assessment and upon completing the baseline data form. We will recruit the participants based on the registration order of women with the clinic, and no other factor will contribute to participants’ order on the list. Each participant on the list will be assigned a consecutive research identification number according to the order by which they will be registered with the clinic. The first participant on the list will be randomly assigned to the MI intervention group, and the next two participants will be assigned to the IMB and IMB-App interventions, respectively. The fourth participant will be assigned to the control group.

### Concealment mechanism {16b}

Randomization will be performed by a person who will not be engaged in the study and will be blinded to the identity of the participants.

### Implementation {16c}

There will be four arms. The abovementioned interventions will be implemented among the three intervention groups. The control group will only receive usual care.

## Assignment of interventions: blinding

This is not applicable. It is not possible to mask this study because of its nature. To avoid bias in the outcome assessment, research assistants concerned with data collection and/or preparation will be blinded to the allocation of the participants.

### Who will be blinded {17a}

As the RCT is an educational intervention study, blinding of participants and researchers is not possible. To minimize selection bias, we will use randomization.

### Procedure for unblinding if needed {17b}

This is not applicable.

## Data collection and management

### Plans for assessment and collection of outcomes {18a}

The primary outcome of the intervention (mode of delivery) will be measured using a checklist after the childbirth.

The secondary outcomes will be measured by a questionnaire 1 month after the interventions.

#### Questionnaire

The questionnaire contains the women’s demographic and obstetrics history information, self-efficacy [56] in CS, and intention [57]. Demographic and obstetrics history items include age, income, educational level (pregnant women and their spouses), employment status (pregnant women and their spouses), number of births, number of pregnancies, current gestational age, number of live children, history of infertility, history of illness, date of birth, and participating in birth classes.

The questionnaire also consists of 17 items about self-efficacy and one item about intention of the women. To measure women’s self-efficacy, we used the Iranian version of the Childbirth Self-Efficacy Inventory (CBSEI) [56]. The instrument was originally developed by Lowe to measure maternal confidence in coping abilities during labor [58]. Khorsandi et al. examined the psychometric properties of the Iranian version and reported that this version was acceptable, reliable, and valid to measure women’s self-efficacy. Items were Likert-type questions, ranging from 1 to 10. Intention was measured using a single item questionnaire [57]. Several studies have found that single item questionnaires would be sufficient to measure some constructs [59].

### Plans to promote participant retention and complete follow-up {18b}

We will follow the participants until the time of delivery. The pregnant women will be participated in this study after ensuring that they have met the study criteria. They will complete informed consent forms. In the first visit, the baseline measurement will be completed by the researcher. During the second visit, the application will be installed on cell phones of the IMB-App group and its operation will be taught to them.

### Data management {19}

To ensure that the data is correctly entered, a double entry of data will be performed by two different individuals. Pregnant women who refer to the hospital will be registered, by chronological order of selection, and they will be allocated randomly in each group. All data obtained from women will be entered to SPSS software. A code will be defined to identify missing data. After screening the data and preparing the data for statistical analysis, first using descriptive statistics such as frequency tables, means and standard deviation, and bar chart and histogram, the distribution of variables will be checked. The obtained data will be kept strictly confidential and will be stored with secured and restricted access; also, the signed consent will be kept locked. Follow-up data are received using the same questionnaires as used at baseline.

### Confidentiality {27}

Only researchers will have access to the personal data in the trial. These data will not be published, and they will be discarded after the publication of results.

### Plans for collection, laboratory evaluation, and storage of biological specimens for genetic or molecular analysis in this trial/future use {33}

This is not applicable. Researchers confirm that there are no laboratory and storage of biological specimens for genetic or molecular analysis in this study.

## Statistical methods

### Statistical methods for primary and secondary outcomes {20a}

Data will be analyzed using descriptive statistics (mean, frequency, and standard deviation); inferential statistics including independent *t* test, paired *t* test, chi-squared, and one-way ANOVA tests; and logistic regression modeling to examine the factors affecting the women’s choice on the mode of delivery to examine the simultaneous effect of variables on the chances of choosing CS. The significance level of the tests will be considered as less than 0.05.

### Interim analyses {21b}

This is not applicable.

### Methods for additional analyses (e.g., subgroup analyses) {20b}

This is not applicable.

### Methods in analysis to handle protocol non-adherence and any statistical methods to handle missing data {20c}

This is not applicable.

### Plans to give access to the full protocol, participant-level data, and statistical code {31c}

Information from the full protocol will be published in a peer-reviewed journal. The relevant data analyzed during the development of this study protocol are available upon request from the corresponding author.

## Oversight and monitoring

A team from Tehran University of Medical Sciences and a peer reviewer (audit) from Iran National Science Foundation (INSF) will regularly monitor the study implementation and datasets and make recommendations on necessary protocol modifications or termination of all or part of the study.

### Composition of the coordinating center and trial steering committee {5d}

This is not applicable.

### Composition of the data monitoring committee, its role and reporting structure {21a}

Data monitoring will be coordinated by INSF. The auditors will follow a monitoring plan to verify that the clinical trial is conducted and that data are generated, documented, and reported in compliance with the protocol and the applicable regulatory requirements.

### Adverse event reporting and harms {22}

Due to the nature of the intervention, there is no adverse event and no harm in this study. Based on our knowledge, the study will not have any negative consequences.

### Frequency and plans for auditing trial conduct {23}

Every 6 months, we will send a report to the auditor.

### Plans for communicating important protocol amendments to relevant parties (e.g., trial participants, ethical committees) {25}

This is not applicable.

### Dissemination plans {31a}

We will share the results of this study with key stakeholders via presenting in related seminars and publishing in peer-reviewed journals. We will also provide and share monographs with related departments in the Ministry of Health, Iran.

## Discussion

This study aims to assess the effect of IM, IMB, and IMB-App on choosing the mode of delivery in pregnant women. Iran has one of the highest CS rates in the region and the world [60]. In line with increasing unnecessary CS, Iran’s healthcare system was reformed and NVD was on the agenda [19]. Many policy interventions have been performed to reduce unnecessary CS rate [39, 40, 61]; however, they were not sustained for a long time. These experiences indicate that the interventions have not been effective enough. Designing appropriate interventions for promoting NVD and decreasing the use of unnecessary CS is one of the most important WHO recommendations on non-clinical interventions on CS [41].

A large portion of increased unnecessary CS rates in Iran is related to women [26, 31, 34, 35, 62–65]. Several studies have reported women’s important role in increasing CS. Women have some problems when they want to choose the mode of delivery. Several studies have shown that the most common reasons underlying the preference for CS in Iran were deep-rooted fear of labor pain and vaginal birth [26, 34, 66–70], irreversible damage to the body and sexual function [23, 28, 35, 36, 70, 71], safety (mother/ baby) and comfort [20, 28, 33, 34, 63, 72], social convenience of birthing to time (time scheduling) [26, 34, 68–70, 73], religious beliefs [28, 34, 70, 72], and cultural beliefs (having role models; modernity, the capability to do vaginal birth) [20, 63, 72, 74, 75]. We realized that most of the reasons come from their beliefs. Therefore, we need to conduct cognitive intervention to decrease the rate. According to the WHO recommendation, psycho-education interventions may be useful to decrease this trend [41].

Due to the target group and their problems with long-term intervention or attendance at classes, we will use brief intervention. The brief intervention is efficacious in mechanisms of change [76–78]. Among different models and techniques, we supposed MI and IMB models can be an effective method of facilitating behavioral changes in CS. The effectiveness of these models has already been confirmed. There are several examples of successful interventions about this technique in health. Rubak et al.’s review has shown that MI is a scientific setting that effectively helps clients change their behavior [79]. The review has shown that MI can be effective even in brief encounters of only 15 min [79]. Another review indicates the potential strength of the IMB model as a theoretical framework to develop behavioral interventions [80]. In this study, we will use both techniques as our study framework for face-to-face education. In recent years, massive smartphone applications have been used widely and effectively in education [81]. We have created a mobile app based on the IMB model to use in a group of interventions. The content has been designed according to the constructs of IMB model.

### Limitations and strengths

We understand that women may be inclined to respond in certain ways because they come to receive certain services. However, we will ensure that their responses will not affect the service they will receive and ensure confidentiality. The self-report nature of the questionnaire and necessity of owning a smartphone are another limitation.

Diverse interventions will allow the researchers to obtain different perspectives from the outcome. We have designed evidence-based non-clinical interventions to reduce unnecessary CSs targeted at women. Causes of increasing CS rates are various and different between and within countries. Before implementing any intervention on the issue, we should recognize the reasons behind this increase, as well as locally relevant determinants of CS, and the views and cultural norms of women and healthcare providers. Our study is supported by evidence-based (qualitative synthesis and meta-analysis in Iran) (unpublished paper) information. We will also use a mobile app, a technology that is ubiquitous and has no limitations. Another strength is the simple and non-expensive nature of the interventions.

## Conclusion

To the best of our knowledge, this paper is the first published paper that describes the protocol of CS with this design (face-to-face and M-health) in Iran. We believe that our study will lead to decrease unnecessary CS in the health system of Iran, and conducting this intervention can increase NVD. To achieve this goal, a systematic review study is done to assess the reason of CS in Iran.

## Trial status

The recruitment of participants for this study started in December 2019 and is still ongoing. The recruitment is expected to be completed in March 2020.

Current protocol version and date: V2.0, 1 December 2019.

## Abbreviations

CS: Cesarean section
NVD: Normal vaginal delivery
WHO: World Health Organization
TUMS: Tehran University of Medical Sciences
INSF: Iran National Science Foundation
